# Feasibility and Assessment of a Cascade Traceback Screening Program (FACTS): Protocol for a Multisite Study to Implement and Assess an Ovarian Cancer Traceback Cascade Testing Program

**DOI:** 10.3390/jpm11060543

**Published:** 2021-06-11

**Authors:** Anna DiNucci, Nora B. Henrikson, M. Cabell Jonas, Sundeep Basra, Paula Blasi, Jennifer Brown, Edward D. Esplin, Dina Hassen, Jing Hao, Yirui Hu, Tracey Klinger, Ilene Ladd, Kathleen Leppig, Meredith Lewis, Michelle Meyer, Steven Ney, Arvind Ramaprasan, Katrina Romagnoli, Zachary Salvati, Aaron Scrol, Rachel Schwiter, Leigh Sheridan, Brinda Somasundaram, Pim Suwannarat, Jennifer K. Wagner, Alanna K. Rahm

**Affiliations:** 1 Mid-Atlantic Permanente Research Institute, 2101 E. Jefferson St, Rockville, MD 20852, USA; Anna.J.DiNucci@kp.org (A.D.); Cabell.Jonas@kp.org (M.C.J.); Sundeep.S.Basra@kp.org (S.B.); Jennifer.L.Brown@kp.org (J.B.); Brinda.X.Somasundaram@kp.org (B.S.); Pim.Suwannarat@kp.org (P.S.); 2 Kaiser Permanente Washington Health Research Institute, 1730 Minor Ave, Seattle, WA 98101, USA; Nora.B.Henrikson@kp.org (N.B.H.); Paula.R.Blasi@kp.org (P.B.); Kathleen.A.Leppig@kp.org (K.L.); Arvind.Ramaprasan@kp.org (A.R.); Aaron.Scrol@kp.org (A.S.); Leigh.E.Sheridan@kp.org (L.S.); 3 Invitae Corporation, 1400 16th St, San Francisco, CA 94103, USA; ed.esplin@invitae.com; 4 Geisinger Department of Population Health Sciences, 100 N. Academy Ave, Danville, PA 17822, USA; dahassen@geisinger.edu (D.H.); yhu1@geisinger.edu (Y.H.); 5 Geisinger Department of Epidemiology and Health Services Research, 100 N. Academy Ave, Danville, PA 17822, USA; jhao@geisinger.edu; 6 Geisinger Genomic Medicine Institute, 100 N. Academy Ave, Danville, PA 17822, USA; tlklinger1@geisinger.edu (T.K.); igladd@geisinger.edu (I.L.); mwlewis1@geisinger.edu (M.L.); smney@geisinger.edu (S.N.); kmromagnoli@geisinger.edu (K.R.); zsalvati@geisinger.edu (Z.S.); rgschwiter1@geisinger.edu (R.S.); 7 Geisinger, Center for Translational Bioethics & Health Care Policy, 100 N. Academy Ave, Danville, PA 17822, USA; mmeyer@geisinger.edu (M.M.); jwagner1@geisinger.edu (J.K.W.)

**Keywords:** implementation, implementation research, ovarian cancer, traceback cascade testing program, genetic testing, micro-costing, HIPAA

## Abstract

Ovarian cancer (OVCA) patients may carry genes conferring cancer risk to biological family; however, fewer than one-quarter of patients receive genetic testing. “Traceback” cascade testing —outreach to potential probands and relatives—is a possible solution. This paper outlines a funded study (U01 CA240747-01A1) seeking to determine a Traceback program’s feasibility, acceptability, effectiveness, and costs. This is a multisite prospective observational feasibility study across three integrated health systems. Informed by the Conceptual Model for Implementation Research, we will outline, implement, and evaluate the outcomes of an OVCA Traceback program. We will use standard legal research methodology to review genetic privacy statutes; engage key stakeholders in qualitative interviews to design communication strategies; employ descriptive statistics and regression analyses to evaluate the site differences in genetic testing and the OVCA Traceback testing; and assess program outcomes at the proband, family member, provider, system, and population levels. This study aims to determine a Traceback program’s feasibility and acceptability in a real-world context. It will account for the myriad factors affecting implementation, including legal issues, organizational- and individual-level barriers and facilitators, communication issues, and program costs. Project results will inform how health care providers and systems can develop effective, practical, and sustainable Traceback programs.

## 1. Introduction

### 1.1. Rationale

Ovarian cancer (OVCA) is the deadliest gynecologic malignancy and the sixth leading cause of cancer death [1,2,3]. In the US in 2017, it was estimated that 233,364 individuals were living with OVCA [1]. When found early, the relative survival rate of OVCA is over 92% [4,5]; however, between 2008–2017, only 16% of US cases were identified at a localized stage. The majority of OVCA cases (58%) were diagnosed at metastatic stages [1].

Germline mutations in *BRCA1* and *BRCA2* genes are a significant risk factor for OVCA [6]. One study reported a 72% lifetime risk of breast cancer and a 44% lifetime risk of OVCA for females with *BRCA1* mutations and lifetime risks of 69% for breast cancer and 17% for OVCA for females with *BRCA2* mutations [7]. Using genetic testing to identify individuals with genetic risk for these associated cancers is critical [8,9,10]. Although genetic testing is recommended for individuals with a personal history of OVCA, one study showed that only around one-third of OVCA patients underwent genetic testing; genetic testing rates were even lower for Black patients [11,12].

Furthermore, since *BRCA* mutations are inherited in an autosomal dominant pattern, the first-degree relatives of OVCA patients have a 50% chance of inheriting the mutation. It is recommended that at-risk relatives be referred for *BRCA* testing (termed “cascade testing”); however, the uptake among eligible relatives is low—only about 20–30%, [13] even when there is no charge to family members for cascade testing [14]. Therefore, more effective genetic testing processes for OVCA patients and relatives is needed to identify individuals with known genetic risk, understand the full scope of clinical needs, and be able to offer the appropriate monitoring and clinical options [15,16].

Numerous barriers at the system, provider, and patient levels impede the uptake of genetic testing among OVCA patients and their relatives. At the system level, several questions remain regarding the real-world feasibility, acceptability, effectiveness, and costs of Traceback screening programs given the current structure of the US health care system and its national and state privacy laws [17,18,19]. At the provider level, a lack of provider knowledge about testing, or an inability to convey the importance of testing, may hinder testing [12]. From the patient’s perspective, an individual’s lack of knowledge/understanding of the importance of testing, communications that do not resonate, sociodemographic factors, family relationships, privacy/discrimination concerns, insurance coverage/costs, and medical mistrust have all been noted as barriers to genetic testing [5,15,16,18,20,21,22,23,24,25,26]. For at-risk relatives, additional barriers exist: there are no standardized best practices for relative identification and cascade testing, and the state and federal privacy laws about genetic information disclosure vary and may hinder familial notification [17,18]. These barriers lower testing rates among both individuals with OVCA and their relatives, and gaps in testing widen by race/ethnicity [12].

A National Cancer Institute (NCI) workshop proposed the Traceback framework as a means of creating a standardized testing process where none previously existed [13,18]. The framework includes three phases to facilitate the identification and genetic counseling for inherited *BRCA* mutations: (1) proband identification, (2) proband testing, and (3) cascade testing of at-risk relatives [13]. The funded study described here offers a systematic assessment of barriers and facilitators to genetic testing, for individuals with OVCA and relatives, to advance Traceback testing in real world clinical settings.

### 1.2. Guiding Framework

The Traceback framework is a staged model involving patients, family, providers, and systems to increase identification of individuals with hereditary cancer risk. In 2016, international experts in genetics, medical and gynecological oncology, clinical psychology, epidemiology, genomics, cost-effectiveness modeling, pathology, bioethics, and patient advocacy convened with NCI scientists to develop the three-phase Traceback cascade testing framework. The Feasibility and Acceptability of Cascade Traceback Screening (FACTS) study will use the Traceback framework to identify OVCA patients who were not previously referred for current standard genetic testing and offer genetic testing for the familial risk for breast or ovarian cancer to patients and their relatives [12]. Our overall project goal is to explore the potential for a comprehensive Traceback program to improve the identification of individuals with a hereditary cancer risk within a health care system. Our project is therefore informed by the Conceptual Model for Implementation Research, which provides guidance for measuring outcomes across multiple levels [27]. The model also both distinguishes between and links implementation processes and outcomes and will inform data collection and interpretation of results across all aims. Our long-term goal is to establish Traceback programs that are generalizable, practical, and sustainable.

### 1.3. Objectives and Aims

FACTS is a prospective observational feasibility study to determine the acceptability, feasibility, and effectiveness of a Traceback cascade testing program in multiple populations and health systems. Our specific aims are:

**Aim 1:** Evaluate a legal solution to facilitate Traceback cascade testing across states and health systems, including examining the potential to use the Public Health Exception for the Health Insurance Portability and Accountability Act (HIPAA) at the federal and state level and reviewing privacy laws that impact the notification of family members.

**Aim 2**: Engage stakeholders to determine culturally appropriate language and communication strategies to facilitate the genetic testing of OVCA patients and relatives.

**Aim 3:** Describe the uptake, effectiveness, and feasibility of a Traceback program in three health systems serving different populations.

3a. Phase I Proband identification: Identify previously diagnosed individuals without current standard genetic testing to measure and compare outcomes at the proband, organization, and population levels.
3b. Phase II Genetic testing of probands: Offer genetic testing to individuals identified in Aim 3a using the culturally appropriate language and communication strategies identified in Aim 2 to measure and compare clinical outcomes at the proband, family member, organization, and population levels.
3c. Phase III Traceback cascade testing of relatives: Approach individuals from Aim 3b with a genetic result related to hereditary cancer using Aim 2-derived language and strategies to encourage cascade testing of relatives to measure and compare clinical outcomes at the proband, family, organization, and population levels.

**Aim 4:** Explore the implementation, service, and clinical outcomes related to a Traceback program at the proband, family member, provider, system, and population levels as guided by the Conceptual Model for Implementation Research to examine the contextual barriers and facilitators of a Traceback cascade testing program [13,27].

## 2. Methods and Design

### 2.1. Overall Study Design and Outcomes

**Figure 1:**
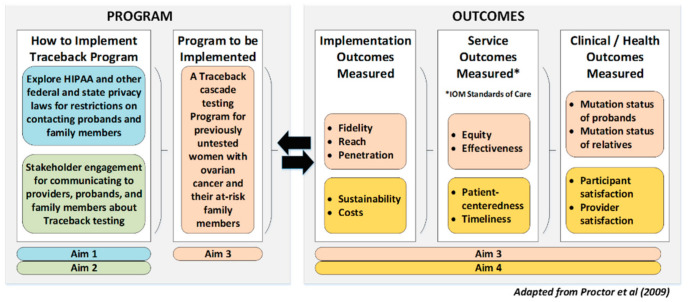
FACTS study conceptual model: adapted from Proctor et al.’s (2009) conceptual model of implementation research.

We will conduct a multisite prospective, observational, feasibility study of the implementation, service, and clinical/health outcomes expected from a Traceback program (Figure 1). The FACTS study conceptual model is based on the model put forth by Proctor et al. (Appendix A) [27]. This is a mixed methods study utilizing legal, micro-costing, qualitative, and quantitative methodologies. We will apply standard legal research methods to examine how federal and state privacy laws affect notification and reporting of genetic test results or genetic risk information (Aim 1). We will conduct semi-structured phone/video interviews with OVCA patients and relatives to determine preferences for communication strategies about Traceback genetic testing (Aim 2); participants will select a phone or video interview according to their preferences. Lastly, we will implement and evaluate the differences, similarities, and adaptations of Traceback (Aims 3 and 4) across three geographically and demographically diverse study sites to guide recommendations for broader research into the implementation of Traceback programs.

### 2.2. Study Setting

The study sites are Geisinger, Kaiser Permanente Mid-Atlantic States, and Kaiser Permanente Washington, representing geographic diversity, racial and ethnic diversity [28], diversity of insurance model, and the opportunity to offer genetic testing to biologically related individuals (Table 1).

### 2.3. Genetic Counseling and Testing Infrastructure

All three sites have clinical geneticists and/or genetic counselors on staff. Standard practice at each site is to offer genetic counseling and subsequently provide multi-gene panel testing as part of usual clinical care (Table A1—Appendix B). Genetic counselors collect a three-generation pedigree, explain the genetic result, provide a letter with a copy of the result and description, and provide a letter describing what the genetic test result means for relatives. Probands are encouraged to share this letter with relatives. The letter mentions the availability of free cascade testing (provided as a standard program by the testing laboratories) to relatives within a specified limited time period regardless of insurance coverage or residence.

### 2.4. Methods by Aim 

Aim 1: Legal solution to facilitate Traceback cascade testing

The primary outcome for Aim 1 is a description of each state’s privacy law. The secondary outcome is guidance for the public health exception to facilitate cascade testing. A separate manuscript will be published in the future that details the methods behind data collection and data analysis for this aim.

Aim 2: Determine culturally appropriate language and communication strategies to facilitate Traceback cascade testing

*Data collection.* Aim 2 will engage stakeholders in discussion to determine culturally appropriate language and communication strategies about genetic testing for individuals with OVCA and Traceback cascade testing of relatives. Aim 2 will also explore other barriers and facilitators to Traceback programs (Table 2). One-on-one interviews and engagement groups will be conducted with patients and community members from each site, using semi-structured interview guides and trained moderators (interview guide available upon request). Participants will receive a $25 incentive.

*Data analysis.* The primary outcomes for Aim 2 are population-appropriate language and communication strategies to inform individuals with OVCA and their relatives about and encourage uptake of genetic testing. This language and these strategies will be incorporated into Aim 3 materials. Secondary outcomes include preferences for language and communication strategies between health systems and populations as well as differences/similarities in language, communication strategies, or other potential barriers and facilitators of Traceback programs.

We will use a thematic analysis approach for the qualitative analysis [29]. Each site will use Rapid Analysis to code focus group and interview transcripts. Through the application of a pragmatic coding scheme, team members will scan transcripts according to a list of modality types. Each modality type will constitute a domain. As each domain is mentioned in the transcript, themes will be derived according to the most common ideas pertaining to said domain. The analysis team will maintain an explanatory description and any exemplar quotes for each theme. In an iterative process, the themes present in transcript summaries will be consolidated by participant type. Similar themes will survive the consolidation process. Any differences or contrary statements will also be recorded.

Aim 3: Describe the uptake, effectiveness, and feasibility of a Traceback Program

*Data collection.* Aim 3 will describe the uptake, effectiveness, and feasibility of a Traceback program in three health systems serving different populations (Table 3). Aim 3′s sub-aims align with the phases of Traceback (a) Phase I: proband identification; (b) Phase II: proband testing; and (c) Phase III: Traceback cascade testing.

3a. Phase I Proband identification. Each site will identify individuals with OVCA or a history of OVCA who have either never had genetic testing or have not received the current standard of genetic testing (Table A1—Appendix B) [30]. Probands will be reviewed to ensure genetic testing eligibility based on ovarian tumor type/histopathology and clinical guidelines.
3b. Phase II Genetic testing of probands. Upon identification of eligible probands in 3a, including their age, tumor type/histology, and date of diagnosis, each site will contact probands using the Aim 2-derived language and communication strategies to offer genetic testing (Table 2). Genetic counseling and testing will be performed per standard clinical protocols using available genetic test panels at each site (Table A1—Appendix B).
3c. Phase III Traceback cascade testing of relatives. Upon proband identification, standard clinical processes for cascade testing will be followed using Aim 2-derived language. All sites will make probands aware of the free family testing available and its time limit. The study team will receive de-identified data from the laboratories on the number of relatives tested per study proband and whether the relative tested positive or negative for the familial variant.

*Data analysis.* Descriptive statistics will summarize testing uptake and genetic mutation rates. Linear and logistic regressions will explore the significant differences between sites, populations, and cancer stage, age, and other demographic variables. Data on the proband and relatives will be described in total and by site. We will use mean/standard deviation or median/interquartile range to summarize continuous variables; we will use frequency and percentage to summarize categorical variables. To create crude comparisons across groups, we will use Analysis of Variance (ANOVA), Kruskal–Wallis, and Pearson Chi-square tests as appropriate.

Aim 3a will enumerate the number of individuals with ovarian cancer within each system (Traceback Phase I). The number and percentage of those with and without a prior genetic result will be summarized and compared across systems using the Pearson Chi-square test. Any differences found to vary across systems (e.g., age, stage, tumor type/histopathology) will be considered as potential confounding variables; a logistic regression model will be adjusted for these variables to estimate the unbiased effects of system differences. The adjusted percentages and odds ratios (ORs) with 95% confidence intervals (CIs) will be presented, with the emphasis on the estimate of the effect rather than the significance level. Similarly, we will summarize the number of women in the sample who are living and still receiving care in their respective system regardless of genetic testing status, compare this number across systems, and use a logistic regression model to adjust for potential confounding. Lastly, we will use the Kaplan–Meier method to estimate the survival curve. Aim 3b will examine the genetic testing uptake among living eligible probands and other outcomes as listed in Table 4 (Traceback Phase II). We will also examine the identification of other hereditary cancer mutations in this population. Since the denominator will change for Aim 3b to include only those successfully tested, we will repeat the summary of variables across all sites and use logistic regression to adjust for potential confounding variables.

Aim 3c focuses on measuring similar outcomes in relatives. We will follow the same summary and modeling of the outcomes as described for Aim 3b.

Aim 4: Evaluate Traceback program outcomes using the Conceptual Model for Implementation Research

*Data collection.* Aim 4 will focus on implementation outcomes to explore the feasibility, acceptability, sustainability, and cost of a Traceback program for ovarian cancer using the Conceptual Model for Implementation Research (Figure 1) [27]. These qualitative and quantitative data will be combined and reviewed with Aim 3′s quantitative outcomes, Aim 1′s legal information, and Aim 2′s engagement information to determine the dissemination potential of a Traceback cascade testing program.

Using a phenomenologic-experiential lens, the study team will invite OVCA patients identified as probands in Aim 3a and their relatives identified in Aim 3c to participate in semi-structured, in-depth qualitative interviews 120 days after the proband genetic test (Table 5). Interviews will assess the core implementation outcomes across all sampling groups, including the acceptability of proband identification; knowledge, attitudes, and beliefs about OVCA and genetic testing; the acceptability of language and strategies used for contact; the logistical barriers and facilitators; the ethical and legal concerns and issues encountered; the insurance issues faced; and any other concerns. Participants will receive incentives.

Physicians and genetic counselors at each site who care for individuals with ovarian cancer will also be invited to complete an interview. Provider interviews will focus on the same implementation outcome domains as the patient interviews. *Data Analysis.*

Program barriers and facilitators. The primary outcome for Aim 4 is the identification of the contextual barriers and facilitators of proband identification and Traceback cascade testing across different health systems and by demographic. This will be a combination of qualitative and quantitative outcomes to understand the experience of individuals and the impact on the feasibility and acceptability of Traceback as a program. The qualitative analysis will use Aim 2′s Rapid Analysis technique.

Program costs. We will apply decision analysis and a simulation modeling approach and utilize a bottom-up micro-costing approach to estimate the costs of each step of a comprehensive Traceback cascade program from each health system’s perspective. Model parameter values will rely on site data, including billing information, supplemented by scientific literature, clinical expertise, and additional sources. The results of this quantitative micro-costing will include total costs of a comprehensive Traceback cascade program and cost per proband with ovarian cancer or cascade testing case identified. All models will be developed transparently using recognized methodological and reporting standards to support generalizability and rigor [31].

## 3. Discussion

### 3.1. Innovation

The FACTS project will generate information useful for health care providers to design and implement Traceback testing programs for OVCA and other conditions. FACTS includes an in-depth legal analysis of HIPAA privacy laws, which will inform how relatives may or may not be contacted, as well as culturally appropriate messaging and outreach strategies to reach target populations. FACTS will offer insight into how to operationalize and incorporate resources such as the time-sensitive family testing options offered by nationwide laboratory companies into a cohesive Traceback cascade testing program. From a clinical perspective, this study will provide critically needed data on the prevalence of cancer predisposition gene mutations (*BRCA1*, *BRCA2*, and others such as *RAD51C, BRIP1, MSH2*) in non-White populations. Ultimately, this study is the first to combine legal, micro-costing, qualitative, and quantitative research with the implementation of science outcomes to provide a comprehensive exploration of best practices for implementing a Traceback program for OVCA patients and their families. The results of this study could also encourage an increase in genetic testing for OVCA genes.

### 3.2. Dissemination of Study Results

We will prepare written summaries of the findings and detailed legal interpretation for Aim 1 for diverse audiences (the public, the scientific community, and the legal/policy community) and for genomics professional organizations and networks. Findings will be disseminated through the Health Care Systems Research Network (HCSRN), a network of 19 health system research centers, as well as to the larger genomics research community through networks such as eMERGE, IGNITE, and ClinGen. Study results will be published in peer-reviewed journals and shared with operational leaders within health systems who are seeking to replicate the program. Tools and learnings created from the micro-costing, engagement activities, and the assessment of implementation outcomes will be consolidated as appropriate into the guidance and toolkits to be disseminated.

### 3.3. Potential Limitations

There are potential limitations to the project described here. First, the Traceback model includes several proposed methods for proband identification to improve the notification of at-risk relatives, including using pathology samples from deceased ovarian cancer patients as a source of genetic information; this project omits that approach. Once the regulatory components and the feasibility of a Traceback program are outlined as described above, future work may explore the use of pathology samples followed by outreach to at-risk family members. Second, the number of interviews planned is insufficient to capture the experiences and preferences of individuals and relatives from gender minority groups (including transgender men, transmasculine individuals, non-binary, and gender-nonconforming people with ovaries); however, these individuals exist in our populations, and Aims 3 and 4 will identify reach and uptake in such individuals. A follow-up study is warranted to explore the preferences for outreach and communication within these groups. Third, this project is taking place amid the COVID-19 pandemic, which will result in unintended but necessary changes to the study approach. The project team is tabulating changes due to COVID-19 and will incorporate that information into the study findings.

### 3.4. Summary and Impact

Few studies have examined the implementation outcomes of cascade testing, and this project addresses this critical unmet need [16,18,27,32,33]. The goal of the funded study outlined here is to determine the feasibility and acceptability of a Traceback program’s three phases in the real-world context of three health systems with different populations, including legal issues, communication strategies, and cost. The project results will inform the development of an effective OVCA Traceback program that is practical and sustainable in real-world settings. The findings could also inform cascade testing processes for other cancers, such as of the colon and endometrium [15,18].

## Figures and Tables

**Table 1:** Overview of participating healthcare systems and genetic screening programs.

					Genetic Counseling & Testing Infrastructure	
Health Care System	Clinical Site (State)	Health Care Delivery Model	Race/Ethnicity All of Patients Served	Added Value of Site	Institution Staff	Testing Vendor
Geisinger	Geisinger (PA)	Open (Geisinger member, other insurance, no insurance)	5% Black, 90% White, 1% Asian, 5% Native Hawaiian/other Pacific Islander, 4% Other, 2% Unknown. 5% Hispanic (Hispanic ethnicity is reported separately).	Includes rural, medically underserved, low-income Multigenerational families	5 Genetic Counselors	Invitae
Kaiser Permanente (KP)	KP Washington (WA)	Closed (KP members only)	6% Black, 72% White, 11% Asian, 1% Native Hawaiian/ other Pacific Islander, 1% American Indian/Alaska Native, 4% Other, 4% Unknown. 6% Hispanic (Hispanic ethnicity is reported separately).	25 full-service clinics in 17 cities	1 Geneticist 5 Genetic Counselors	Invitae
Kaiser Permanente (KP)	KP Mid-Atlantic States (D.C, MD, VA)	Closed (KP member only)	36% Black, 25% White, 12% Asian, 0.4% Native Hawaiian/other Pacific Islander, 0.2% American Indian/Alaska Native, 1% Other, 24% Unknown. 12% Hispanic (Hispanic ethnicity is reported separately).	Substantial racial/ethnic diversity	2 Geneticists 7 Genetic Counselors	Invitae

**Table 2:** Stakeholder groups and discussion topics explored in Aim 2 sessions.

Stakeholder Group	Discussion Topics for All Stakeholder Groups
Individuals with personal history of ovarian cancer Relatives (i.e., individuals with a family history of ovarian cancer) Community Advisory Groups	Modes of contact and messages to ovarian cancer patients who have not had genetic testing or have not had the current standard of genetic testing Modes of contact and messages to encourage the cascade testing of relatives Modes of contact and messages for community-based outreach to raise awareness about genetic testing for ovarian cancer patients and relatives of ovarian cancer patients Barriers and facilitators to the Traceback approach

**Table 3:** Aim 3 inclusionary criteria.

	Probands	Relatives
Inclusion criteria	Receiving care at FACTS study sites Age ≥ 18 years Personal history of ovarian/peritoneal/fallopian cancer diagnosis from 1980–present Alive at recruitment Ability to complete consent process in English	Family history of one or more 1st or 2nd degree adult relative with a history of ovarian, peritoneal, or fallopian cancer * Alive at recruitment
Exclusion criteria	Receiving hospice care Confirmed previous receipt of current standard genetic testing	Receiving hospice care Confirmed previous receipt of current standard genetic testing Personal history of ovarian, peritoneal, or fallopian cancer

* determined by medical records where possible, otherwise self-report.

**Table 4:** Aim 3 quantitative outcomes.

Traceback	Sub-Aim	Primary Outcome	Secondary Outcome
Phase I: Proband identification	3a	Baseline Fidelity to guidelines: Number in registry with known genetic result	Baseline equity: demographic differences in diagnosis, age, stage, tumor type/histopathology, prior cancer, survival by healthcare system, and race/ethnicity
		Baseline Reach: Number in registry living and still receiving care in system	
Phase II: Proband genetic testing	3b	Reach: eligible vs. tested probands	Rate of positive, negative, VUS for BRCA1 and BRCA2
		Fidelity: eligible women who received the notification of testing availability	Rate of positive, negative, VUS for other cancer risk genes
		Effectiveness: uptake of testing and eligible probands	Differences in mutations and rates by age, stage, tumor type/histopathology, race/ethnicity, and other demographics
		Equity: differences in uptake by healthcare system, age, stage, tumor type/histopathology, race/ethnicity	
Phase III: Family member identification and testing	3c	Reach: eligible family members informed by probands	Rate true positives and negatives for BRCA1 and BRCA2
		Effectiveness: uptake of testing by eligible family members	
		Equity: differences in uptake by healthcare system, age, stage, race/ethnicity	Rate of true positives and negatives for other cancer risk genes.

**Table 5:** Aim 4 data collection.

Interview Sample	Number
Providers	5 per site
Probands who did not have testing	10 per site
Probands who tested (positive or negative)	5 per site
Family members who had cascade testing	5 per site
No cascade testing	10 per site

## Appendix A

Adapted by permission from Springer Nature Customer Service Centre GmbH: Springer; Administration and Policy in Mental Health and Mental Health Services Research; Implementation Research in Mental Health Services: an Emerging Science with Conceptual, Methodological, and Training challenges, Proctor, E.K.; Landsverk, J.; Aarons, G.; Chambers, D.; Glisson, C.; Mittman, B. © 2021 Springer Nature Switzerland AG (2009).

## Appendix B

**Table A1:** Hereditary cancer genes tested by commercial lab panel.

Invitae Clinical Panel Breast-Gyn Panel
ATM BARD1 BRCA1 BRCA2 BRIP1 CDH1 CHEK2 DICER1 EPCAM MLH1 MSH2 MSH6 NBN NF1 PALB2 PMS2 PTEN RAD50 RAD51C RAD51D SMARCA4
